# The Tobacco Heart

**Published:** 1901-04-06

**Authors:** 


### THE TOBACCO HEART.

Among the various causes by which the action of the heart may be disturbed tobacco occupies an important place. It is of especial interest in that the effect of tobacco can be studied unhampered by many of the complicating circumstances which tend to make the investigation of some other cardiac poisons, such, for example, as alcohol, so difficult. Alcohol undoubtedly poisons the heart, but at the same time it produces a widespread effect on all the tissues, often an actual degeneration, so that in studying the operation of alcohol on the heart we are in most cases studying its action in a diseased organism. But in tobacco we have a poison which shows its effect upon hearts still sound, and in constitutions otherwise apparently uninjured, and we are thus able to inquire how far a merely temporary toxic agent may be efficient in producing symptoms of cardiac disease. In the Lettsomian Lectures, recently delivered, Dr. Mitchell Bruce says that the uncomplicated effects of tobacco on young healthy hearts, as they present themselves clinically, are palpitation in every instance, a sense of irregular action, post-sternal oppression and pain in half the cases, and in one out of every eight sufferers either angina or uncomfortable sensations in the left arm. Faintness or actual faints occur in one-third, and giddiness and a feeling of impending death in a smaller proportion. In 50 per cent. of the cases the heart appears to be of the ordinary size, in a few it is very slightly enlarged; the præcordial impulse is often very weak, but occasionally increased in force and frequency, and almost as often irregular as not; the pulse tension is nearly always low. Out of twenty such cases complaining of the heart, not one presented a cardiac murmur beyond a weak systolic bruit varying with posture or cubitus. So much for the cardiac symptoms produced by tobacco in the presumably healthy heart. Now let us turn to the man over forty. In such cases, while palpitation is still the common complaint, pain including angina is put forward more prominently, and so are faintness, actual faints, a feeling of impending death, and a sense of cardiac irregularity, each intermission being accompanied with a sudden stab through the præcordia. In these subjects the heart is more frequently found to be large and feeble, the same weak systolic murmur as has been mentioned above is to be heard occasionally, the radial pulse is often irregular, and the vessel-wall is thick. Thus we have a combination of symptoms and signs sufficient to alarm the casual observer. But if we study them in the light of what we know about the tobacco heart in young subjects on the one hand, and of what we know about the normal condition of the heart and arteries at sixty years of age on the other hand, we may be able to reassure ourselves. Every cardio-vascular change which is found in great smokers must not be put down to tobacco, but *vice versâ* præcordial pain, angina, faintness, and irregular pulse, in a man 60 years of age with a full-sized heart, are not to be hastily regarded as evidences of grave disease without further inquiry as to his habits. The cardiac enlargement and large pulse may be nothing more than the result of a life of bodily and mental activity; the præcordial distress and the other symptoms may be the result only of tobacco. Dr. Mitchell Bruce relates several cases in which well-marked angina was clearly the result of tobacco, and ceased to trouble when the tobacco was given up. The moral of this is important, and extends far beyond the question of the tobacco heart, for if tobacco can by itself do so much damage, and if in cases of cardiac disease, or when, without the actual presence of disease, the heart is merely getting older, the addition of tobacco poisoning can produce so marked a simulacrum of severe cardiac disease (all of which may vanish on removing this superadded cause) we must in all cases of heart disease look out for the superadded and possibly remediable cause by which the present condition has been produced, and not be content with ascertaining the more or less permanent disorder with which the heart has been more or less successfully struggling perhaps for a long time. We are all apt, says Dr. Mitchell Bruce, to overlook in diagnosis, and still more in treatment, the fact that, whether in an ordinary senile heart, or in a heart that is the seat of chronic valvular disease, or in arterial degeneration, something more than the pathological changes has to to be regarded—usually some entirely adventitious disturbance which alone calls for treatment, such as indigestion, flatulence, worry, a bronchial catarrh, or it may be free indulgence in tobacco, tea, or coffee.

